# Awareness of both global uncertainty and feedback in human time estimation

**DOI:** 10.3758/s13414-025-03115-5

**Published:** 2025-07-09

**Authors:** Chetan Desai, Farah Bader, Martin Wiener

**Affiliations:** Department of Psychology, George Mason University, 4400 University Drive, Fairfax, VA 22030 USA

**Keywords:** Time perception, Feedback, Learning, Temporal error monitoring

## Abstract

Recent behavioral studies have shown that humans possess self-awareness of their individual timing ability in that they can discern the direction of their timing error. However, in these studies which included a single repeat (re-do) trial for each duration, it remains unclear whether the reduction in errors in the re-do trials was due to self-awareness of individual timing ability or because the participants used the feedback from the initial trials to improve on the re-do ones. To investigate this further, we conducted a behavioral study in which subjects were divided into two groups: one in which the “re-do” phase occurred frequently, but not always (80% of trials; called the “high-double” group), and one in which re-do trials were rare (20% of trials; called the “low-double” group). This was done to test the possibility of subjects relying on the re-do trials as a method of improvement. Subjects significantly improved in their performance on re-do trials regardless of whether re-dos were rare or frequent. Further, an unexpected finding was observed, where subjects in the low-double group also overall performed better than those in the high-double group. This finding suggests that subjects, knowing that re-do opportunities were rare, engaged better timing at the outset; yet these subjects still improved on re-do trials, suggesting humans are able to incorporate both global uncertainty and feedback.

## Introduction

The ability to incorporate sensory input, especially the perception of the passage of time, toward compensatory goal-directed change in action (error correction), is an essential component of survival in both human and nonhuman animals. Indeed, time estimation has been recently suggested as a fundamental feature of error-monitoring abilities (Fu et al., 2023). Self-assessment of one’s own timing ability without any external feedback, known as temporal metacognition, is vital for reliably determining temporal accuracy and variance despite uncertainty (Akdoğan & Balcı, 2017; Balci et al., 2011; Lamotte et al., 2012). However, previous research on temporal error monitoring has provided mixed results for awareness of timing errors in humans. Initial work demonstrated that humans could detect both the magnitude and direction of their timing errors when asked to provide reports on their own performance (Akdoğan & Balcı, 2017; Kononowicz et al., 2019). Yet other studies suggested that human subjects could only determine if they had made an error, not the direction of the error (Brocas et al., 2018; Riemer et al., 2019). A critical aspect of human awareness between these experiments relates to the nature of feedback provided. Feedback can be provided in a variety of ways for timing experiments—it can be adaptive or nonadaptive, absolute (correct/incorrect), directional (early/late), or some combination of the above. A critical issue, however, is whether subjects have the opportunity to apply their feedback to update their representations and improve their performance (Ryan, 2016). Previous studies have used interval timing tasks to investigate this issue where participants are instructed to estimate temporal intervals of several seconds (Buhusi & Meck, 2005). One particular task, temporal reproduction, involves exposure to a specific duration (encoding) and then an opportunity to recreate the interval duration via key press (reproduction), with or without feedback. Previous research has shown that human subjects can accurately infer the distribution of intervals presented, and use this information to guide reproductions in the face of measurement uncertainty (Acerbi et al., 2012; Jazayeri & Shadlen, 2010). While the evidence already points toward an existing self-awareness of time, evaluating timing aptitude in context of feedback and learning may broaden our understanding of internal metacognitive process and its role in time perception.

Commonly, subjects in temporal reproduction studies exhibit a pattern of central tendency in which the shortest intervals within the stimulus set are overestimated and the longest intervals are underestimated. Contrary to expectations, the performance of subjects in temporal reproductions tasks do not seem to improve with feedback where subjects still exhibit a central tendency pattern, even when informed of the nature of their errors. Instead, feedback only serves to reduce a general tendency to over- or underestimate (Ryan, 2016). Notably, on any given trial, subjects were unaware of the interval that will be tested, and so feedback from a previous trial could not be applied to the next one, or worse, erroneously applied (Glasauer & Shi, 2022). To rectify this issue, a recent study introduced a “re-do” version of the temporal reproduction task, in which the interval tested is random, but each trial has a single repeat, such that subjects can attempt to reproduce the interval a second time (Bader & Wiener, 2021). Additionally, subjects received nondirectional adaptive feedback that notified them only whether the reproduced interval fell within a proportion of the presented interval, but not whether they were early or late. Remarkably, subjects improved both their accuracy and precision, as measured by the coefficient of variation (CV), on the re-do trials, such that the central tendency effect was attenuated and the CV was smaller. Further, a second group of subjects on the re-do trials was tested without any feedback provided. Here, again, an attenuation of the central tendency effect was observed, but with no concomitant improvement in precision. This second finding suggests that humans have intrinsic access to their timing errors (Kononowicz & van Wassenhove, 2019) but require feedback to specifically reduce the variability of their timing. This finding mirrors recent work in nonhuman primates where reinforcement improves sensorimotor timing precision (Wang et al., 2020), which is implemented via afferent fibers in the ventrolateral thalamus projecting to the supplementary motor area (SMA).

To examine how brain networks interact during timing and error monitoring in humans (Bader & Wiener, 2024) carried out a neuroimaging study where subjects performed the re-do time reproduction task as described above but with all subjects receiving feedback after every trial while undergoing functional magnetic resonance imaging (fMRI). Behaviorally, the researchers replicated the effect found in their previous study, with subjects reducing their error on the re-do trials, such that the central tendency effect was significantly reduced. Planned contrasts comparing performance between the estimation and reproduction phases, as well as initial and re-do trials revealed a notable dissociation in which brain network was invoked between these two phases and trial types. Specifically, default mode network (DMN) activation was more prevalent during the estimation phase, when subjects were encoding the presented interval, whereas the timing network (TN) was more active during the reproduction phase, when subjects were reproducing that interval. DMN structures included regions such as the precuneus, anterior cingulate cortex, superior frontal and angular gyri, as well as the posterior cingulate cortex, whereas TN regions included the SMA and left precentral gyrus, as well as regions of the visual cortex. Between trial types (initial and re-do), network activation shifted again, but in different directions; in particular, the DMN became more active during re-do trials in the estimation condition, when subjects were incorporating feedback to update their representation, whereas the TN became less active during re-do trials in the reproduction condition. Both cases involved a greater/lesser extension of the network being activated, such that more DMN regions and fewer TN regions were active; indeed, the SMA was the only remaining structure in the reproduction re-do phase.

Notably, in the re-do tasks described above, all subjects received feedback on both initial and re-do trials. This leaves open the possibility of subjects relying on the re-do trials as a method of improvement. That is, since the re-do trials occurred with 100% probability in the fMRI-EEG study, subjects may have intentionally performed poorly on the initial trials so as to use the feedback to improve on the re-do trials. This raises the question of how “automatic” the awareness of errors is in temporal error monitoring. To determine this, we present here an experiment in which human subjects performed the re-do time reproduction task as described above and in our previous studies (Bader & Wiener, 2021, 2024). However, in the present experiment, the re-do trial was probabilistic; that is, subjects were not assured of getting a re-do opportunity on every trial. Instead, we divided subjects into two groups: one in which the re-do trials occurred on 80% of all trials (termed “high double” to reflect the high likelihood of a re-do opportunity on any given trial), and one in which re-do trials occurred on 20% of all trials (termed “low double” to reflect the low likelihood of a re-do opportunity on any given trial).

**Fig. 1:**
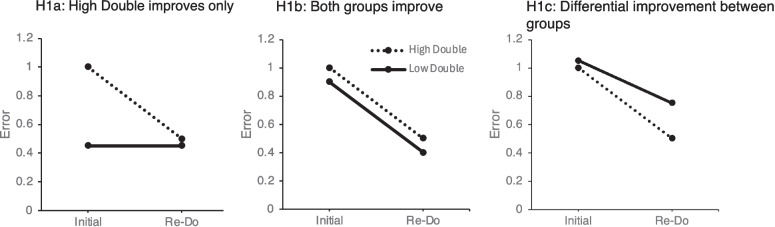
Three hypothetical scenarios for differences between high- and low-double groups. In H1a, the high-double group shows a reduction in error rate, but the low-double group does not, reflecting a lack of awareness of errors. In H1b, both groups show improvement, reflecting awareness of errors regardless of re-do opportunities. In H1c, both groups improve, but the low-double group shows less improvement, reflecting awareness of errors, but a lack of preparation to incorporate those errors into future performance

We outline three hypothetical outcomes between both groups (Fig. 1); across all three, we predicted the high-double group would show the same results as our previous two reports, with a decrease in errors on re-do trials compared with Initial trials. For the low-double group, our first hypothesis (H1a) is that the low-double group will show no improvement on re-do trials relative to initial trials. Under this hypothesis, low-double subjects are unprepared for the re-do opportunity and so are not metacognitively self-monitoring their performance. In the second hypothesis (H1b), both groups show a similar level of improvement on re-do trials, reflecting the possibility that subjects are able to metacognitively self-monitor regardless of whether they will be given a second chance or not. In the third hypothesis (H1c), both groups show improvement on re-do trials, but the low-double group does not show as much improvement as the high-double group; this hypothesis represents the possibility that the low-double group is unprepared for the re-do opportunity yet still retains some metacognitive awareness of their prior performance. Across all three outcomes, we made no specific predictions regarding between-group differences in baseline levels of performance; for example, in H1a, the low-double group may or may not perform better on the initial trials than the high-double group, reflecting the possibility that the high-double group intentionally performs worse on initial trials as they are aware of the high likelihood of a re-do opportunity.

## Methods

### Subjects

Forty subjects were recruited for this experiment; however, for the final analysis, 37 right-handed and neurologically healthy subjects (average age = 20.81 ± 4 *SD*, 28 men, nine women) were included. Two subjects were unable to finish the entirety oSf the behavioral experiment, while for another participant, there was a data loss due to a technical issue related to the computer. Nineteen subjects were assigned to the low-double group (average age of 21 ± 4.58 *SD*, 14 men, five women). Eighteen subjects were assigned to the high-double group (average age of 20.6 ± 3.5 *SD*, 14 men, four women).

An independent *t* test revealed that the ages between the low-double and the high-double were not significantly different, *t*(35) = 0.291, *p* < 0.772, CI [− 2.321, − 3.098]. The Pearson’s chi square, $\chi$ ^2^(1) = 0.084, *p* = 0.772, for the gender ratios was also not significantly different between the Low Double and High Double groups.

### Task paradigm

The temporal reproduction task was delivered via Psychopy2 on a 27-in. Mac desktop while subjects sat 60 cm from the computer screen (Dell S2716DGR, 120 Hz refresh rate). The task structure was comprised of three phases: estimation, reproduction, and feedback.

Depending on the type of trial, the three phases could be performed once (initial, single trial) or twice (initial + re-do, double trial) for each duration. There were two experimental conditions and subjects could be randomly assigned to either of the two groups that had varying ratios of double (initial + re-do) and single (initial only) trials. Subjects were blinded to the group assignment and did not know prior to an upcoming trial whether it would be a single or double trial. The low-double group comprised 80% of total trials with only one opportunity (single, initial only) to perform the trial. The other 20% of total trials in the low-double group consisted of both an initial and re-do trial (double). Conversely, in the high-double group, 80% of the total trials had a second chance (double; initial + re-do) whereas the other 20% of the total trials were single trials. Both low-double and high-double conditions had 250 trials total, five blocks and breaks following each block.

**Fig. 2:**
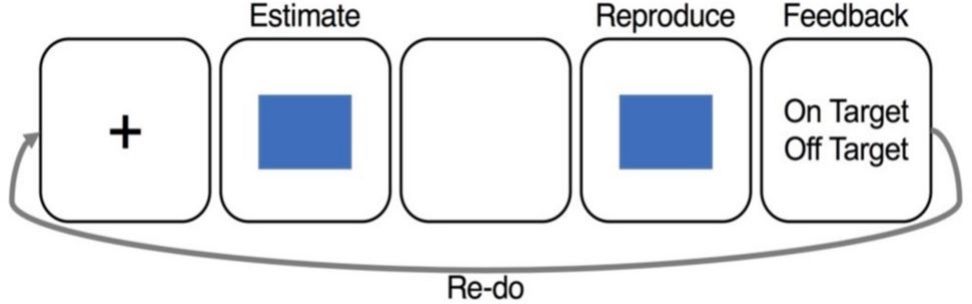
Task schematic for the stages of the task (encoding, reproduction, feedback) and the “re-do” included in a “double” trial. A blue square is displayed for a randomized duration from 1.5–6 s and disappears on its own. A second blue square then appears and the participant must press a number key to make it disappear once the on-screen duration time matches. Feedback indicating whether the participant was on-target or off-target is then deliver*ed. The participant then has a second opportunity to re*-do the trial in its entirety, with a second recurrence of the estimation and reproduction stimulus, another key press response to terminate the reproduction stimuli, and a post response feedback. *Note that a* “single” trial with only an initial trial would not include a “re-do” of all the task stages. (Color figure online)

Each trial initiated with a centrally presented fixation cross for a randomly presented duration of 2–6 s. In the estimation phase, a blue square was visually shown to the participant for one of five logarithmically spaced, randomly presented intervals (1.5–6 s). Until the square was on-screen, the participant was instructed to encode the duration in memory and to not use counting as a method to do so, which has been demonstrated as an effective means of eliminating counting strategies (Rattat & Droit-Volet, 2012). Following the estimation phase, there was a 4–8-s gap prior to the reproduction phase. Then, the blue square reappeared on-screen in the reproduction phase, and the participant was asked to press any number key when the blue square had remained on-screen for the same time duration as the time elapsed in the estimation phase. This key press caused the square to disappear, signaling interval termination (Fig. 2).

After every trial, adaptive feedback (duration = 1 s) was delivered 2–4 s after the disappearance of the square and informed the participant whether the response was on target or off target; notably, this feedback provided no index of direction (i.e., early or late). On each trial, the response was on target if it fell within a specified window around the target duration. This window was scaled with the reproduced interval by a feedback constant (*k*), starting with an initial value of 3.5. The value of *k* was adjusted such that the reproduced interval had to be within the range [interval/*k*] and was updated according to the 1-up/1-down rule with a step size of 0.015 which was either added or subtracted for each correct or miss trial (Jazayeri & Shadlen, 2010). If the participant’s reproduced interval was either 0.015 above or below the target duration, an on-target feedback would be delivered; otherwise, an off-target feedback was delivered. For example, for the first trial in the session with a target duration of 6 s, the on-target window would be [4.286, 7.714] obtained by subtracting and adding 1.714, which is the ratio of the target = 6 s and *k* = 3.5. If the reproduced duration falls within this interval for a correct trial, on-target feedback would be delivered. For the next trial, 0.015 will be added to the value of *k* from the previous trial and the procedure above would be repeated to produce the new narrower target window. *Critically, in the double trials participants had a second opportunity (essentially a re-do trial) to perform the entire sequence of phases (estimation, reproduction, and feedback) again, ensuring feedback was applied to the appropriate duration.* Participants were not informed prior to the beginning of any trial whether they would have one (single) or two (double) opportunities (an initial and a re-do trial) to time the visual stimulus (blue square) which would be of the same duration in both types of trials. In the double trials, subjects were able to distinguish the initial from the re-do because in the re-do trials, the text would read “Estimate-again” and “Reproduce-again.”

### Statistical analysis

Jeffreys’ Amazing Statistics Program (JASP, Version 0.16; c) and R (Version 4.4.0) were used to analyze the behavioral data from the two conditions (low-double and high-double) temporal reproduction task. The data were normally distributed and passed the Shapiro–Wilk test of normality for the proportional temporal error, accuracy, and precision [as measured by the coefficient of variation (CV)]. Temporal error was calculated as the absolute difference between the reproduced duration and the target duration. The CVs were calculated as the standard deviation of the reproduced durations divided by the participant’s mean reproduced durations and two separate sets of CV values were generated for the initial and re-do trials. The slope was calculated from a regression line of the mean reproduced durations against the sample target durations. Two slope values representing the initial and re-do slopes were generated. A generalized linear mixed effects model of error was used to detect changes between initial and re-do trials. This approach was chosen due to the unequal numbers of trials between initial and re-do in high-double and low-double groups. The model included two fixed effects terms (trial type: initial and re-do; group type: high-double and low-double) as categorical effects. For the random effects structure, we adopted the complex random intercepts approach advocated by (Scandola & Tidoni, 2024), which included subject and subject:trial type as random effects. Following this procedure, we initially designed a linear mixed-effects model, but observed that the residuals were not normally distributed, due to the right-skewed distribution of error values. We found that a generalized linear model with a Gamma distribution and identity link function exhibited normally distributed residuals; model fits were additionally not singular and did not fail to converge, along with normally distributed random effects. Outlier values that were three median absolute deviations from the median were removed using the MATLAB function “isoutlier” before model fitting was performed. We chose this measure, as opposed to the mean, as it is more robust when dealing with skewed distributions (Leys et al., 2013), as are often observed in reaction time and time reproduction experiments (Wilcox & Rousselet, 2018).

## Results

**Fig. 3:**
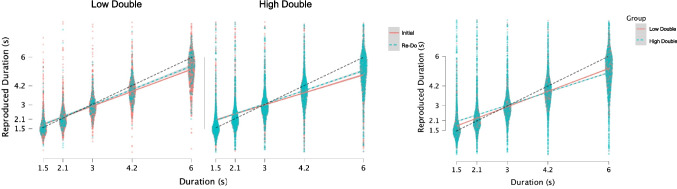
Differences in reproduction performance between groups. Panels display individual trial data from all participants. Left two panels display reproduction performance separately for Low and high double groups for initial and re-do trials, while right panel displays the average performance across trial types between groups. Dashed lines indicate the identity, where perfect performance would lie. All subjects exhibited central tendency effects, with a reduction on re-do trials, yet the low-double group exhibited a lesser central tendency than the high-double group. (Color figure online)

**Fig. 4:**
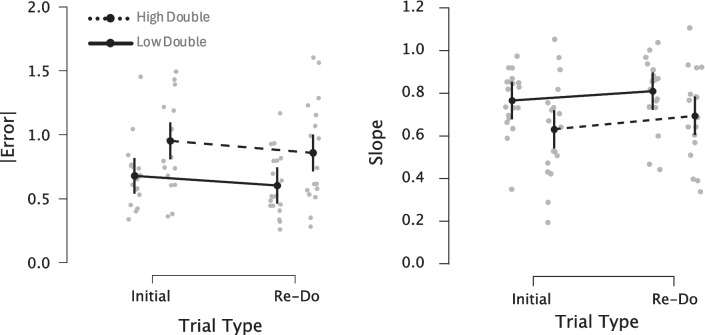
Differences between groups in accuracy of reproduction performance. *Left panel depicts the mean absolute temporal error (in seconds) across intervals on* initial and re-do trials. *Both groups exhibited a significant reduction in error on* re-do trials compared with initial ones, but with no difference in the level of improvement between groups. *Additionally, the* low-double group exhibited significantly lower error globally compared with the high-double group. *Right panel displays slope values from a simple linear regression of mean reproduced durations against presented ones; values closer to 1 indicate veridical performance. Again, both groups exhibit improvements on* re-do trials, but with no difference between groups in the level of improvement, as well as globally better performance for the low-double group. *Error bars represent 95% confidence intervals; gray points represent individual subjects*

The results of the generalized linear mixed effects model revealed a main effect of trial type, such that error was lower on re-do trials compared with initial ones ($\beta$= *− *0.07501, *SE* = 0.02197, *t* = − 3.414, *p* = 0.000641). Additionally, we observed a main effect of group type, with the high-double group exhibiting significantly higher error than the low-double group ($\beta$ = 0.27329, *SE* = 0.10212, *t* = 2.676, *p* = 0.007449). No interaction between trial type and group type was observed $($ $\beta$ = − 0.01856, *SE* = 0.03022, *t* = − 0.614, *p* = 0.539), suggesting that both groups improved to a similar degree (Fig. 3). For slope values, a repeated-measures analysis of variance (ANOVA) similarly demonstrated a main effect of trial type, *F*(1,35) = 8.748, *p* = 0.006, ${\eta }_{p }^{2}$= 0.2, and of group type, *F*(1,35) = 4.488, *p* = 0.041, ${\eta }_{p}^{2}$ = 0.114, but no interaction, *F*(1,35) = 0.286, *p* = 0.596, with slope values increasing on re-do trials to a similar degree between groups, but with overall higher slope values for the low-double group (Fig. 4).

**Fig. 5:**
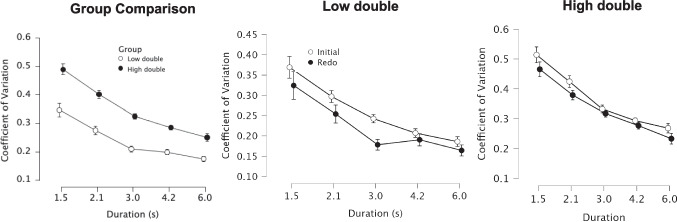
Changes in CV *between groups and trial types. Left panel displays a comparison between groups, collapsed across trial type, in which the* low-double group exhibited significantly lower CVs across all tested durations. *Right panels display the change in CV across trial type for each group, separately. Both groups exhibited lower CVs on* re-do trials compared with initial ones. *Error bars represent within-subject standard error*

For the CV analysis, a repeated-measures ANOVA, with trial type and duration as within-subjects factors, and group type as a between-subjects factor, revealed a main effect of trial type, *F*(1,35) = 19.678, *p* < 0.001, ${\eta }_{p }^{2}$= 0.36, with lower CVs on re-do compared with initial trials, as well as a main effect of group type, *F*(1,35) = 8.659, *p* = 0.006, ${\eta }_{p}^{2}$ = 0.198, with overall lower CVs for the low-double group compared with the high-double group. A main effect of duration was also observed, *F*(1.847,102.166) = 61.217, *p* < 0.001, ${\eta }_{p}^{2}$ = 0.636, with lower CVs for longer intervals. No interactions between any of the variables were observed (all *p* values > 0.05), again indicating a similar level of change between trial types in each group (Fig. 5).

## Discussion

Our study demonstrated the beneficial impact of the opportunity to correct errors in a single re-do trial following feedback from the initial trial regardless of whether these opportunities were rare or frequent. In support of hypothesis H1b, temporal estimates improved on the re-do trials similarly between the participants in the high-double and the low-double group for both accuracy and precision. This finding supports the possibility that subjects are able to metacognitively self-monitor their errors, regardless of whether they will be given a second chance or not.

Researchers found that the process of learning interval durations in humans transpires fairly quickly and errors can be minimized within only one trial (Simen et al., 2011). To measure the speed of temporal learning, Simen et al. (2011) devised the “beat the clock task,” a paradigm where subjects must learn to time an unknown interval that can randomly change without warning between sets of trials. It is noteworthy that despite the endogenous timing, uncertainty stemming from the presentation of rapidly changing durations, performance improvement was not impeded (Simen et al., 2011). In fact, all beat the clock participants improved in response times after only a single trial of beat-the-clock task, such that after showing noticeably lower error in the second trial, the subsequent trials did not show any further marked decrease in errors (Simen et al., 2011). This finding is consistent with our current study’s results demonstrating that the performance on the second trial following previous on-target feedback improved the accuracy and precision of temporal estimates. Another study that employed the repeat trial and found similar benefits was developed by Mitani and Kashino (2018) and found similar benefits as to those in Simen et al. (2011). However, in this task, participants were required to reproduce the duration of a single tone after hearing it twice successively without feedback after the initial trial. Feedback was then delivered and participants indicated whether they were early or late in responding. Bias and variability improved, self- judgment of timing error matched the actual temporal reproductions, and serial dependency was dampened but only for subsecond rather than suprasecond intervals (Mitani & Kashino, 2018). Our previous studies (Bader & Wiener, 2021, 2024) extended the findings of this study to the visual modality and demonstrated the benefit of reducing timing variability in the suprasecond range, with our current study further probing the metacognitive awareness of errors in the estimation of temporal intervals.

In addition to demonstrating the beneficial impact of a single repeat trial on temporal estimation following feedback from the initial trial, we found increased sensitivity to longer durations. Regardless of whether it was a re-do or an initial trial and whether it was a high double or a low-double group, participants were more precise in their estimates of longer temporal intervals than shorter ones. This finding violates the Weber’s Law for timing, known as scalar property within the field of interval timing, which predicts that the relative precision of temporal measurement, measured using the CV, should remain constant across a range of intervals (Gibbon, 1977). This prediction implies a constant CV for intervals timed by any single mechanism, but not for intervals timed by different mechanisms. To test this prediction, Lewis and Miall (2009) employed a broad range of intervals (68 ms to 16.7 min) over a visual temporal reproduction task where participants reproduced intervals after receiving feedback from the initial trial. Here, the hypothesis was that the boundaries between the temporal ranges of mechanisms are marked by sudden changes (breakpoints) in relative precision (CV). In support of our finding and in violation of the Weber’s Law for interval timing, the CVs did not appear to remain constant even over a narrower range of durations from 600 to 3,000 ms and demonstrated a smooth logarithmic decrease instead (Lewis & Miall, 2009). Although this finding does not exclude the possibility of multiple mechanisms as the gradually decreasing curve could represent an envelope function containing a number of overlapping mechanisms with similar CVs, it raises the possibility of a single mechanism used across a range of durations (Lewis & Miall, 2009). Notably, although this study tested participants over a broad range of intervals, the finding held even over a narrower range of intervals similar to those used in our study.

Findings from several neuroimaging studies support a single network-based mechanism for timing that is functionally distinct from that associated with the metacognitive awareness of errors (Wiener et al., 2010). Results from our previous fMRI study generally align with the present study and our understanding of the network-based mechanisms associated with timing and metacognitive awareness of errors. This study found both a disassociation between the activation of regions in the DMN and those in the TN and a double dissociation in the functional connectivity between these regions across the initial and re-do trials and across the estimation and reproduction phases (Bader & Wiener, 2024). Specifically, while estimating a duration was more likely to invoke DMN regions, reproducing a duration was more likely to invoke timing regions. This suggests that whereas subjects are actively aware of time’s passage when explicitly encoding a time interval into memory during estimation, the motor system drives the transformation of that estimate into a timed action when reproducing the interval (Bader & Wiener, 2024). Also, this study found that internetwork communication between the DMN and the TN increased more when estimating a time interval on initial trials and when reproducing one on re-do trials. One possible reason for this difference could be that on initial trials, subjects may already be aware of their error in their initial response, and so only require network cross-talk again when reproducing the interval again on the re-do trial, when subject performance improved (Bader & Wiener, 2024). Altogether, the results from our current study are consistent with these findings where improved performance on the re-do trials could be explained by learning of the time interval that is facilitated by DMN during the initial trial and then transformed into timed action by TN when reproducing that interval during the re-do trial.

Our study has a few limitations that should be noted. We found that subjects in the high-double group tended to be less accurate than those in the low-double group. Furthermore, low-double group subjects were also more precise in their estimates than those in the high-double group regardless of whether it was the initial or the re-do trial. This, in contradiction with hypothesis H1c, suggests that subjects in the low-double group, knowing that the re-do opportunities were rare, engaged better timing at the outset, but were also metacognitively aware of their errors to the same extent as those in the high-double group to improve their performance on the re-do trials. To further evaluate this claim, it may be necessary to query subjects on the metacognitive awareness of their errors during the initial trial.

## Conclusion

Overall, our study deepens our understanding of the role of metacognitive awareness of errors in temporal estimation and the effect of learning on these errors in conditions where second opportunities to improve (re-do trials) are either rare or frequent events. Further, it suggests that humans may be automatically aware of their own timing errors, regardless of whether these errors will be used to improve immediate performance or not. Lastly, it suggests that, when humans are aware of the chance to improve, they can adjust their own timing (un)certainty to perform at a level with greater accuracy and precision.

## Data Availability

All data and materials are available online (https://osf.io/vntwd/).

## Code availability

Available upon request.

## Declarations

### Open practices statement

The data and materials for all experiments are available online (https://osf.io/vntwd/).
